# Clinicopathological alterations in naturally occurring *Babesia gibsoni* infection in dogs of Middle-South Gujarat, India

**DOI:** 10.14202/vetworld.2017.1227-1232

**Published:** 2017-10-14

**Authors:** Avinash K. Bilwal, Ghanshyam C. Mandali, Falguni B. Tandel

**Affiliations:** Department of Veterinary Medicine, College of Veterinary Science and Animal Husbandry, Anand Agricultural University, Anand, Gujarat, India

**Keywords:** babesiosis, clinicopathological changes, dogs, intraerythrocytic piroplasm

## Abstract

**Aim:**

The present research work was undertaken to describe various clinical signs and hematobiochemical alterations in dogs affected with *Babesia gibsoni*.

**Materials and Methods:**

Blood smears from a total of 79 suspected dogs of Anand region as well as Surat region of Gujarat state (India) were screened for detection of intraerythrocytic piroplasm of small form of *Babesia*. Diagnosis was made on the basis of clinical signs and demonstration of *B. gibsoni* organism in Giemsa-stained thin blood smears. The clinical signs were recorded at the time of presentation, and blood samples were subjected to estimation of hematobiochemical parameters by auto hematology analyzers at College of Veterinary Science and Animal Husbandry, Anand. Statistical analysis, interpretation, and comparison of hematobiochemical changes with scientific literature were carried out to understand the pathophysiology of the disease.

**Results:**

Out of 79 dogs, 16 were positive for naturally occurring babesiosis based on the presence of intraerythrocytic piroplasm of small form of *Babesia* in blood smears. The clinical cases were manifested by wide variety of non-specific clinical signs. The hematological evaluation revealed that the mean values of hemoglobin and total erythrocyte counts in dogs with babesiosis decreased significantly (p<0.01) in comparison to healthy dogs. Among differential leukocyte count, mean values of neutrophils and eosinophils increased while lymphocytes decreased (p<0.01) in dogs with babesiosis in comparison to healthy dogs. Serum biochemistry revealed increase (p<0.01) value of alkaline phosphatase, alanine aminotransferase, aspartate aminotransferase, and globulin as well as decrease in albumin levels (p<0.05) in dogs with babesiosis as compared to healthy dogs.

**Conclusion:**

*B. gibsoni* is having multisystemic effects with atypical hematobiochemical changes in dog are discussed here, which would aid new insights in diagnosis of disease.

## Introduction

Blood-feeding ectoparasites such as ticks, fleas, sand flies, and mosquitoes can transmit many dangerous pathogens to dogs - such as bacteria, protozoa, viruses, or helminths. They may lead to a variety of serious infections, mostly classified by their vectors: Tick-borne diseases, flea-borne diseases, sand fly-borne diseases, and mosquito-borne diseases [1]. Tick-borne hemoparasites are one of the most important vector-borne infections of dogs. They are numerous and are caused by several etiological agents such as bacterial, protozoan, and rickettsial organisms [2]. Among them, tick-borne hemopathogens such as Babesia, Ehrlichia, Anaplasma, Borrelia, and Hepatozoon are of major health concern to dogs and some of which are of zoonotic significance [3]. Canine babesiosis is a clinically significant and geographically widespread hemoprotozoan disease of domesticated dogs and wild canids [4]. The commonly occurring *Babesia* species in dogs are the *Babesia canis* and *Babesia gibsoni* [5]. Based on genetic data, vector specificity and variation of pathogenicity, large *Babesia* are subdivided into three species, namely, *B. canis* transmitted by *Dermacentor reticulatus* (in Europe), *B. vogeli* transmitted by *Rhipicephalus sanguineus* (in tropical and subtropical regions), and *B. rossi* transmitted by *Haemaphysalis elliptica* (in South Africa). Small *Babesia* includes *B. gibsoni* (Asian genotype), *Babesia conradae* (previously Californian genotype), and *Babesia microti* like species (also referred to as the Spanish isolate or *Theileria annae*) [6]. The disease has been reported in various states of India including Gujarat [7-10]. The life cycle of *B. gibsoni* includes two stages: Inside the host red blood cells (RBCs), in which the sporozoites convert into piroplasms and the other inside the tick vector [11]. A small form of *Babesia* is seen in Giemsa-stained peripheral blood smear of small 1-3 µm in diameter, ring-, oval-, or comma-shaped piroplasms suggestive of *B. gibsoni* which can be confirmed by polymerase chain reaction [12]. The parasite also transmitted by blood exchange and transplacental route can also be possible [13]. The immunological response plays the most important role in pathogenesis of canine babesiosis. These parasites initiate a mechanism of antibody-mediated cytotoxic destruction of circulating erythrocytes. Autoantibodies are directed against components of the membranes of infected and uninfected erythrocytes which causes intravascular and extravascular hemolysis [14,15].

The clinical-pathological changes, including hematology and blood chemistry, are nonspecific. The various clinical symptoms regularly depend on the severity of the disease in infected animals. The typical clinical findings include anemia, thrombocytopenia, leukocyte abnormalities, increased liver enzymes, and hyperbilirubinemia. hypokalemia, hyperglobulinemia, azotemia, metabolic acidosis, and abnormalities of urinalysis may be observed in some severely affected dogs [16]. Supportive treatment is usually given and includes fluid therapy, anti-inflammatory and antipyretics, gastroprotectants, oxygen supplementation, and blood transfusion should be employed when necessary.

The naturally occurring cases of *B. gibsoni* are having variety of clinical manifestations ranging from anorexia to hepatomegaly or splenomegaly or death making it difficult to have a definitive diagnosis solely on the basis of clinical examination. Hence, the study was conducted to describe various clinical signs and hematobiochemical alterations in dogs affected with *B. gibsoni*.

## Materials and Methods

### Ethical approval

Samples were collected from clinical cases coming to Teaching Veterinary Clinical Complex (TVCC), Veterinary College, Anand Agricultural University, Anand and Nandini Veterinary Hospital, Surat. Hence, this particular study did not require ethical approval.

### Sample collection

Privately owned dogs presented to TVCC, Anand and Nandini Veterinary Hospital, Surat, Gujarat (India), were examined clinically irrespective of their age, breed, and sex from July 2015 to March 2016. Dogs showing clinical signs suggestive of babesiosis were screened out during period and among that total 79 peripheral blood smears were made from suspected dogs and examined for the presence of intraerythrocytic piroplasms. Dogs presented to clinics for checkup which are healthy, from ectoparasites dewormed, and vaccinated were used as healthy control group, and blood was collected from them aseptically. Diagnosis of babesiosis was confirmed by cytological examination of Giemsa-stained peripheral blood smears made from ear tips. A 5 ml of blood samples from positive dogs were collected in a sterile anticoagulant vial containing ethylenediaminetetraacetic acid (K_3_). Hematological parameters were analyzed by auto hematoanalyzer (Analytical, Hema 2062, Analytical Technologies Limited, Vadodara, India). Serum biochemical parameters were analyzed using an auto-biochemical analyzer (Mindray, BS-120 chemistry analyzer) using standard kits manufactured by coral clinical systems auto-chemistry analyzer using commercial diagnostic kits procured from Crest Biosystem (A Division of Coral Clinical System, Goa) at the Department of Veterinary Physiology and Biochemistry, College of Veterinary Science and Animal Husbandry, Anand with standard laboratory protocols. Hematobiochemical changes in dogs with naturally occurring babesiosis were compared with healthy dogs.

### Statistical analysis

The data obtained were subjected to the statistical analysis described by Snedecor and Cochran [17]. The independent t-test having means with unequal variances was carried out. Variables with p<0.05 were considered as statistically “significant,” variables with p<0.01 were considered as statistically “highly significant” and variables with p>0.05 were considered as statistically “non-significant.”

## Results and Discussion

About 16 out of 79 peripheral blood smears were having small single oval piroplasms inside the red blood cells indicative of *B. gibsoni*, so the overall prevalence was 20.25%. Little information in literatures is available on prevalence in Gujarat. Jadhav *et al*. [10] studied the epidemiology of canine babesiosis in Gujarat, and that was 15.81%. The vital signs such as rectal temperature, respiration rate, heart rate, and capillary refilling time were significantly (p<0.01) increased in babesiosis positive dogs than healthy dogs (Table-1).

**Table-1:** Clinical variants (mean±SE values) in healthy and babesiosis positive dogs.

Clinical variant	Healthy dogs	Babesiosis positive dogs
Rectal temperature (°F)	102.00±00.21	102.96±00.23**
Heart rate (beats/min)	117.00±03.04	129.88±02.94**
Respiration rate (breaths/min)	28.50±01.67	36.56±02.22**
CRT (seconds)	01.25±00.16	02.37±00.13**

** p<0.01. CRT=Capillary refill time, SE=Standard error

In this study, rectal temperature was elevated in only 5 (31.25%) cases while remaining (68.75%) of the dogs had near to normal temperature. Thus, it is apparent that pyrexia is not a cardinal sign in canine babesiosis due to *B. gibsoni*. These findings were in agreement with Varshney *et al*. [18] and Yadav *et al*. [19]. The respiration rate, heart rate, and capillary refilling time were increased than healthy dogs may be due to marked anemia and stress [18,20].

### Clinical signs

There were a wide variety of clinical signs were observed at the time of presentation including general state signs (50.82%), gastrointestinal sings (13.94%), cardiac signs (13.11%), nervous signs (13.11%), respiratory signs (4.92%), and urological signs (4.10%). The details of each clinical sign with percentage are presented in Table-2.

**Table-2:** Clinical signs in dogs with babesiosis.

Different forms	*B. gibsoni* n=16 (%)
General state signs	
Ticks on body	15 (93.15)
Weakness/emaciation/apathy	11 (68.75)
Dehydration	9 (56.25)
Recumbancy/prostration	7 (43.75)
Petechiae/epistaxis/hemorrhage	3 (18.75)
Pale mucus membrane	9 (56.25)
Congested	4 (25.00)
Icteric	4 (25.00)
Gastrointestinal signs	
Anorexia	8 (50.00)
Vomiting/nausea	4 (25.00)
Diarrhea	2 (12.50)
Salivation	3 (18.75)
Nervous signs	
Dullness/depression	11 (68.75)
Ataxia/paresis/epilepsy	5 (31.25)
Cardiac signs	
Arrhythmia	10 (62.50)
Weak pulse	6 (37.50)
Respiratory sign	
Nasal discharge/coughing	3 (18.75)
Dyspnea	3 (18.75)
Urological sign	
Dribbling of urine	3 (18.75)
Hemoglobinuria	2 (12.50)

B. gibsoni=Babesia gibsoni

Here, it was seen that 50.82% of the dogs were having clinical signs of general state. The tick infestation was found in 93.15% (15/16) cases.

Godara *et al*. [21] stated that there was a positive correlation between the presence of ticks on the body surface of host and hemoprotozoan infections. Weakness, dehydration, and recumbency were other non-specific clinical signs observed, accordance with Karunakaran *et al*. [22] and Yadav *et al*. [19]. Pale mucous membranes were due to marked anemia and petechiation was due to intravascular hemolysis. Jaundice is one of the most commonly reported complications of canine babesiosis [23]. Anemia is typically due to both intravascular and extravascular hemolysis [24]. Anorexia was evident in 50.0% cases, and other similar, different gastrointestinal clinical findings in *B. gibsoni* positive dogs were also reported by Varshney *et al*. [18], Yadav *et al*. [19], and Kumar *et al*. [25]. Nervous system involvement was observed in 13.11% cases. Cerebral babesiosis occurs with the sludging of erythrocytes within central nervous system capillaries leading to tissue hypoxia, weakness, ataxia, seizures, and vestibular or cerebellar signs [26]. Cardiac arrhythmia and dyspnea may be to pulmonic changes or cardiopulmonary involvement [18]. Hemoglobinuria was observed only in two cases. Unlike bovine babesiosis, hemoglobinuria is rarely seen in canine babesiosis [18,27-29]. Oliguria is an ominous sign in dogs affected with renal impairment due to babesiosis [30]. These findings suggest that there is large variation in clinical signs in canine babesiosis and wide range of inconsistent clinical manifestations might be due to multisystemic effects of disease.

### Hematological parameters

Levels of hemoglobin (Hb), total erythrocyte count (TEC), and packed cell volume (PCV) decreased significantly in babesiosis positive dogs than healthy dogs (Table-3). Decrease in Hb levels were in agreement with reports of Furlanello *et al*. [31], Niwetpathomwat *et al*. [32], Selvaraj *et al*. [8], Shah *et al*. [33], Wadhwa *et al*. [34], Andoni *et al*. [35,36], Reddy *et al*. [37], and Nalubamba *et al*. [38].

**Table-3:** Hematological parameters (mean±SE) in healthy and babesiosis positive dogs.

Parameter	Healthy dogs (n=8)	*Babesiosis* positive dogs (n=16)
Hb (g/dl)	13.23±00.43	09.33±00.80**
TEC (×10^6^/µl)	06.66±00.30	04.80±00.43**
PCV (%)	38.46±01.64	30.26±02.62*
TLC (×10^3^/µl)	12.30±00.91	16.54±02.71
Neutrophils (%)	66.62±01.60	81.72±02.04**
Lymphocytes (%)	29.00±00.75	12.21±02.25**
Monocytes (%)	02.87±00.54	02.06±00.23
Eosinophils (%)	01.50±00.32	04.95±00.85**
Basophils (%)	00.00±00.00	00.00±00.00
Platelet count (×10^3^/µl)	242.25±16.99	246.59±35.68
MCV (fl)	64.64±01.60	64.58±00.92
MCH (pg)	21.50±00.70	22.60±01.08
MCHC (g/dl)	33.05±00.56	31.67±01.01

** p<0.01,

* p<0.05.

TEC=Total erythrocyte count, PCV=Packed cell volume, TLC=Total leukocyte count, MCHC=Mean corpuscular hemoglobin concentration, MCV=Mean corpuscular volume, MCH=Mean cell hemoglobin, SE=Standard error, Hb=Hemoglobin

Decreased TEC levels were in accordance with reports of Niwetpathomwat *et al*. [32], Shah *et al*. [33], Andoni *et al*. [35,36], Reddy *et al*. [37], and Nalubamba *et al*. [38]. Hb and TEC levels could be due to epistaxis, petechial hemorrhages, direct mechanical disruption caused by parasite as it leaves red blood cells, intravascular hemolysis, and immune-mediated or non-immune mediated destruction of red blood cells or due to severe anemia.

The difference between total leukocyte count and platelet count in dogs with babesiosis and healthy were statistically non-significant which was contrast to Shah *et al*. [33] and Sivajothi *et al*. [39] who reported significant decreased platelet counts (i.e., thrombocytopenia). Selvaraj *et al*. [8], Wadhwa *et al*. [34], and Reddy *et al*. [37] reported the presence of significant leukopenia in dogs with babesiosis. Decreased platelet counts in babesiosis were in agreement with reports of Furlanello *et al*. [31], Niwetpathomwat *et al*. [32], Shah *et al*. [33], Wadhwa *et al*. [34], Andoni *et al*. [35,36], Reddy *et al*. [37], Nalubamba *et al*. [38], and Vishnurahav *et al*. [40]. *Babesia* initiates a mechanism of antibody-mediated cytotoxic destruction of circulating erythrocytes and anemia may be more dependent on the host immune response than on the direct destruction of RBC by the piroplasm [41]. The mechanisms of the thrombocytopenia are not yet fully understood in babesiosis. Elevated body temperature could have contributory effect on thrombocytopenia [42]. Decreased PCV levels in dogs with babesiosis were in agreement with reports of Furlanello *et al*. [31], Niwetpathomwat *et al*. [32], Selvaraj *et al*. [8], Shah *et al*. [33], Wadhwa *et al*. [34], Andoni *et al*. [35,36], Reddy *et al*. [37], and Nalubamba *et al*. [38].

Among differential leukocyte count, levels of neutrophils increased significantly in dogs with babesiosis than healthy dogs. The reason might be due to coinfection. These findings were in accordance with reports of Selvaraj *et al*. [8], Shah *et al*. [33], and Vishnurahav *et al*. [40]. However, Reddy *et al*. [37] reported the decreased count of neutrophils in dogs with babesiosis. Levels of lymphocytes decreased significantly in dogs with babesiosis than healthy dogs. Lymphocytopenia may be due to concurrent viral infection associated with babesiosis. These findings were in accordance with reports of Selvaraj *et al*. [8] and Andoni *et al*. [35]. However, Shah *et al*. [33] and Reddy *et al*. [37] reported a significant increase in lymphocytes count in dogs with babesiosis. The differences between monocytes and basophils in dogs with babesiosis than healthy dogs were statistically non-significant. However, Reddy *et al*. [37] reported significant monocytes changes in dogs with babesiosis.

### Serum biochemical parameters

Among various serum biochemical parameters, levels of alkaline phosphatase (ALP) increased significantly in dogs with babesiosis than healthy dogs (Table-4). These findings were in accordance with Shah *et al*. [33]. Increase in level of ALP was may be due to damage or abnormal function of biliary system [43]. Levels of alanine aminotransferase (ALT) increased significantly in dogs with babesiosis than healthy dogs. This present observation simulates to the findings of Wadhwa *et al*. [34] and Reddy *et al*. [37]. Levels of aspartate aminotransferase (AST) increased significantly in dogs with babesiosis than healthy dogs (Table-3). These findings were in agreement with Wadhwa *et al*. [34]. Increased activities of AST and ALT were might be due to escape of these enzymes from the damaged hepatic parenchymal cells with necrosis or altered membrane permeability indicating hepatic dysfunction [28]. Levels of total bilirubin increased significantly in dogs with babesiosis than healthy dogs. These findings were in accordance with Shah *et al*. [33]. Hyperbilirubinemia was due to resulted from both intravascular and extravascular hemolysis [44].

**Table-4:** Serum biochemical parameters (mean±SE) in healthy and babesiosis positive dogs.

Parameter	Healthy dogs (n=8)	Babesiosis positive dogs (n=16)
ALP (IU/L)	78.38±04.15	283.06±30.81**
ALT (IU/L)	34.81±03.20	69.06±05.80**
AST (IU/L)	45.44±04.83	67.55±03.77**
Total bilirubin (mg/dl)	00.40±00.05	00.67±00.08*
BUN (mg/dl)	14.84±00.88	29.96±02.50
Creatinine (mg/dl)	01.03±00.12	00.93±00.10
Total protein (g/dl)	05.90±00.37	05.46±00.46
Albumin (g/dl)	01.72±000.14	02.37±00.20
Globulin (g/dl)	01.47±00.24	03.23±00.38
A:G (g/dl)	01.29±00.15	01.37±00.08

** p<0.01,

* p<0.05.

ALP=Alkaline phosphatase, ALT=Alanine aminotransferase, AST=Aspartate aminotransferase, BUN=Blood urea nitrogen, SE=Standard error

Levels of blood urea nitrogen (BUN) increased significantly in dogs with babesiosis than healthy dogs. Reddy *et al*. [37] also recorded significant increase in level of BUN in babesiosis positive dogs and healthy dogs. Here, only the level of BUN is increased indicative of non-renal influences. This elevation may be due to hemolysis of RBCs, gastrointestinal hemorrhage or blood transfusion. Elevations in serum urea may be caused by a recent protein meal or increased tubular reabsorption during low tubular urine flow rates. A recent protein meal, however, is an unlikely event in canine babesiosis patients because of appetite depression [45]. The difference between values of creatinine in babesiosis positive dogs and healthy dogs were statistically nonsignificant. These findings were in agreement with Wadhwa *et al*. [34]. However, Andoni *et al*. [36], Reddy *et al*. [37], and Vishnurahav *et al*. [40] reported significant increase in creatinine in babesiosis positive dogs and healthy dogs.

Levels of albumin and A:G ratio decreased significantly (p<0.01) in dogs with babesiosis than healthy dogs. These results were in accordance with Yadav *et al*. [19]. Levels of globulin increased significantly (p<0.01) in dogs with babesiosis than healthy dogs. These finding were in agreement with Vishnurahav *et al*. [40] and Vijayalakshmi *et al*. [46]. The difference between values of TPP in babesiosis positive dogs and healthy dogs were statistically nonsignificant. These findings were in accordance with Reddy *et al*. [37].

## Conclusion

The overall prevalence of babesiosis, based on peripheral blood smear examination was recorded as 20.25%. The dogs with naturally occurring babesiosis exhibited clinical signs include tick infestation, emaciation, anorexia, and dullness. Characteristic hematological pattern associated with babesiosis in dogs showed that there was decreased level of Hb, TEC, and PCV as well as increased level of neutrophils while serum biochemical pattern include increased the level of ALP, ALT, AST, total bilirubin, and globulin. During the study, it was observed that *B. gibsoni* had a wide variety of clinical manifestations so dogs are showing erratic fever, weight loss, depression, pale mucosa, and splenomegaly alone or in combination can be suspected for babesiosis. All the suspected dogs in this study were diagnosed only based on the microscopic examination of the stained peripheral blood smears. Microscopic examination may not detect low parasitemia though; it remains the most rapid confirmatory method which was carried out in this study. As the disease is spread through vector tick, control of the vector tick is essential for prevention of disease. Application of ectoparasiticides with acaricidal/insecticidal and additional repellent efficacy reduces the arthropod-host interaction including attachment to the skin and blood feeding and can thus reduce the risk of infection. Prevention of tick attachment and flea or sand fly or mosquito bites must be an established tool of disease prophylaxis in any dog living in vector endemic areas or traveling with its owner to such regions. Dog owners should be made aware of the risks and the need for protection by their veterinarians.

## Authors’ Contributions

The present study was a part of AKB’s original research work which includes experimental design, a collection of blood and serum samples, examination of blood smear, estimation of hematobiochemical parameters and statistical analysis, preparing, and drafting the manuscript during M.V.Sc. thesis program. GCM had designed the plan of work as well as provided guidance during the entire experiment and corrected manuscript. FBT helped during sampling, statistical analysis, and manuscript preparation. All authors read and approved the final manuscript.
